# Managing the Loss of Right Renal Venous Tissue in Deceased‐Donor Organ Procurement

**DOI:** 10.1155/joot/3588832

**Published:** 2026-09-02

**Authors:** Nguefouet Momo Rostand Emmanuel, Riccardo Giuseppe Bertolo, Luigino Boschiero, Alessandro Antonelli

**Affiliations:** ^1^ Departmental Unit of Kidney Transplant Surgery, Department of Surgery and Dentistry, Azienda Ospedaliera Universitaria Integrata di Verona, Verona, Italy, ospedaleuniverona.it; ^2^ Division of Urology, Department of Surgery and Dentistry, Azienda Ospedaliera Universitaria Integrata di Verona, Verona, Italy, ospedaleuniverona.it

**Keywords:** iatrogenic injury, inferior vena cava, renal vein repair, right kidney, venous tissue loss

## Abstract

**Background:**

Procurement‐related injury to the right renal vein (RRV) or renal–caval vein complex (RCVC) may preclude transplantation of an otherwise usable right kidney. We report an expanded single‐center experience with the previously described York Plastic technique, focusing on technical feasibility and short‐term outcomes.

**Methods:**

We retrospectively reviewed deceased‐donor right kidneys assessed at our center between January 2018 and January 2025. Of 334 kidneys, 17 (5.1%) sustained procurement‐related RCVC injury; 15 underwent York Plastic reconstruction and transplantation, whereas 2 were not used because of severe associated graft damage. Of the 15 transplanted cases, 10 were included in our preliminary 2023 technical report and 5 are newly reported.

**Results:**

All 15 reconstructed grafts were transplanted successfully. No intraoperative bleeding, renal vein thrombosis, or Doppler evidence of venous stenosis occurred. Mean bench reconstruction time was 35 minutes (range, 20–51), and mean cold ischemia time was 16.4 ± 2.5 h. Median serum creatinine among functioning grafts was 1.58 mg/dL at 1 month, 1.49 mg/dL at 3 months, and 1.36 mg/dL at 12 months. At 12 months, 13 of the 14 surviving recipients had functioning grafts. One recipient died at 3 months with a functioning graft; one surviving recipient returned to dialysis before 12 months because of antibody‐mediated rejection associated with severe cytomegalovirus infection.

**Conclusions:**

The York Plastic technique appears to be a feasible bench‐salvage option for selected procurement‐related RCVC injuries and may support organ preservation in carefully chosen cases.

## 1. Introduction

Renal transplantation is considered the gold standard treatment for patients with end‐stage renal disease, but organ availability remains a critical issue, and waiting times may extend for years in many allocation systems [1, 2]. A contributing factor to this shortage is discard of donor kidneys because of anatomic abnormalities or procurement‐related injury, particularly for the right kidney [3–5].

One of the main anatomical challenges in using the right kidney for transplantation is its shorter renal vein. This feature complicates procurement and may increase technical difficulty at implantation. In living donation, the risk of graft failure has been reported to be higher when transplanting the right kidney [6, 7]. In deceased‐donor kidney transplantation, the inferior vena cava (IVC) is commonly used to extend the right renal vein (RRV) [5]. However, living‐donor transplantation may require more complex surgical strategies, including extensive recipient vessel mobilization [8] or vascular grafts [9, 10].

Excessive transection of the IVC involving the RRV orifice during procurement from a deceased donor carries a high risk of stricture when closed with a running suture above the left renal vein orifice. A kidney damaged during procurement should therefore be evaluated for possible surgical reconstruction according to graft quality and transplant‐surgeon expertise, rather than being discarded automatically [11].

We report an expanded single‐center experience with the previously described York Plastic technique. Reference [12] used to compensate for iatrogenic venous tissue loss in the renal–caval vein complex (RCVC) during liver and kidney procurement. Unlike the preliminary 2023 AJT report, which described the first 10 reconstructed cases, the present manuscript reports the full institutional experience during the study interval, including 17 kidneys with procurement‐related RCVC injury: 15 reconstructed and transplanted kidneys, of which 5 are additional reconstructed cases not previously reported and 2 are additional injured kidneys that were not reconstructed because of severe associated arterial and/or parenchymal damage. The present study therefore adds broader clinical context, longer follow‐up, a pragmatic injury‐pattern classification, and a more explicit discussion of how this reconstruction fits within bench‐salvage strategies for compromised right kidneys.

## 2. Materials and Methods

### 2.1. Study Design and Case Selection

A single‐center, retrospective observational study was conducted at the Azienda Ospedaliera Universitaria Integrata di Verona. All deceased‐donor right kidneys assessed at our center between January 2018 and January 2025 were screened to identify procurement‐related venous injuries involving the RCVC. Cases eligible for inclusion were those in which the IVC cuff/RCVC was excessively transected during liver–kidney procurement, resulting in a venous defect judged unsuitable for standard caval tubulization or transverse closure because of the high risk of outflow narrowing. During the study period, 334 deceased‐donor right kidneys were assessed; 17 (5.1%) showed procurement‐related RCVC injury. Fifteen were reconstructed with the York Plastic technique and transplanted, whereas 2 were not reconstructed because of concomitant major arterial and/or parenchymal damage that made the grafts unsuitable for clinical use. Of the 15 reconstructed and transplanted kidneys, the first 10 had been reported in our preliminary 2023 AJT technical letter, whereas 5 are newly reported in the present series. To reduce selection and reporting bias, the entire source population was screened, both transplanted and nontransplanted kidneys with RCVC injury were included, overlap with the preliminary report was explicitly disclosed, and outcomes were reported using a standardized 12‐month landmark. Donor and recipient variables, bench reconstruction time, Doppler parameters, cold ischemia time, and clinical outcomes were retrieved from institutional records.

### 2.2. Procurement Context and Injury Pattern

**FIGURE 1:**
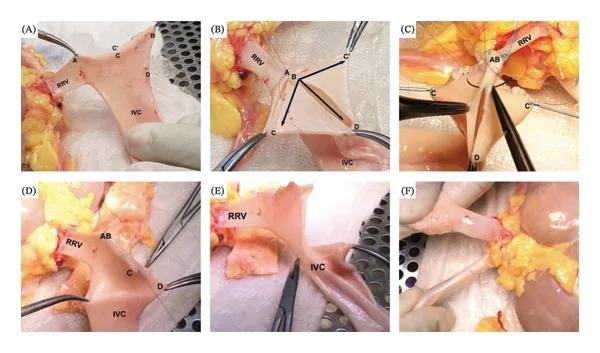
York Plastic reconstruction for Type 1 renal–caval vein complex injury. (A) Type 1 procurement‐related RCVC injury, characterized by excessive transection of the inferior vena cava with preservation of the native right renal vein ostium. The anatomical landmarks *A*, *B*, *C*, *C*′, and D are identified; the *B*–*D* margin corresponds to the residual left renal vein ostium. (B) Points A and B are approximated to create the common upper apex, AB. The arrows indicate the three planned suture directions from AB toward *C*, *C*′, and *D*. (C) The remaining caval flaps are aligned and sequentially sutured along the three limbs of the reconstruction, creating a Y‐configured venous conduit with a broad outflow. (D) Completed Y‐shaped reconstruction before final tailoring, showing the convergence of AB with *C*, *C*′, and *D* and preservation of the venous lumen. (E) Final bench appearance after trimming redundant caval tissue, resulting in the shortest conduit compatible with a tension‐free venous anastomosis and without excessive redundancy or kinking. (F) Bench flushing test performed to confirm conduit patency, suture‐line integrity, and the absence of leakage. Abbreviations: RCVC, renal–caval vein complex; RRV, right renal vein; RRA, right renal artery; IVC, inferior vena cava.

**FIGURE 2:**
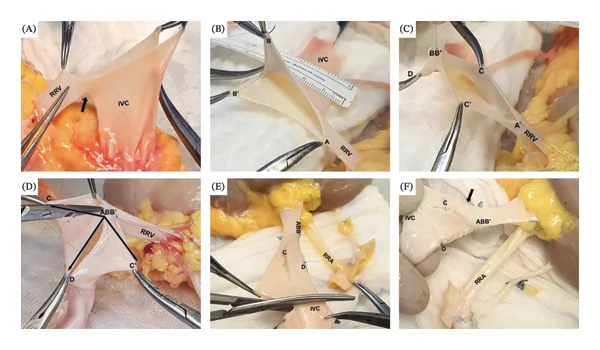
York Plastic reconstruction for Type 2 renal–caval vein complex injury. (A) Type 2 procurement‐related RCVC injury, characterized by excessive caval transection with loss of right renal venous tissue and disruption of the native left renal vein ostial rim. The arrow indicates the anticipated area at risk of stenotic narrowing if conventional closure were performed. (B) The disrupted ostial margins are identified, including points *B* and *B*′, together with point *A* at the residual origin of the right renal vein. (C) Points *B* and *B*′ are initially approximated to reconstruct the disrupted ostial component, forming BB’. Point *A* and the remaining caval flaps *C*, *C*′, and *D* are displayed in preparation for the subsequent reconstruction. The BB′–D margin corresponds to the residual left renal vein ostium. (D) Point *A* is approximated to BB’ to create the common upper apex, ABB’. The arrows indicate the three planned suture directions from ABB’ toward *C*, *C*′, and *D*. (E) Completed Y‐shaped reconstruction before final tailoring. The Y‐configured venous conduit recreates a broad outflow cone while compensating for ostial tissue loss and preserving the venous lumen. (F) Final bench appearance after trimming redundant caval tissue, showing a short, broad, tension‐free conduit without excessive redundancy or kinking and with a satisfactory relationship to the right renal artery. The arrow identifies the reconstructed venous segment corresponding to the area considered at greatest risk of stenosis before reconstruction. Abbreviations: RCVC, renal–caval vein complex; RRV, right renal vein; RRA, right renal artery; IVC, inferior vena cava.

All grafts were recovered using an en bloc abdominal procurement. After the separation of the left kidney from the en bloc specimen, the right graft was delivered with the remaining segment of IVC, the RRV at its confluence, and the renal artery on an aortic patch. Despite satisfactory macroscopic graft appearance, the venous complex showed iatrogenic injury caused by excessive or inappropriate IVC transection during combined liver–kidney procurement. For pragmatic intraoperative decision‐making, RCVC injuries were categorized into two patterns according to the relationship between the transection plane and the RRV ostium. Type 1 injury referred to excessive caval transection without the loss of RRV tissue, with preservation of the native RRV ostium (Figure 1). Type 2 injury referred to excessive caval transection associated with loss of right renal venous tissue and disruption of the adjacent left renal vein ostial rim (Figure 2). This classification was developed as a practical operative framework and has not undergone external validation or formal assessment of interobserver reproducibility.

### 2.3. Bench Reconstruction (York Plastic Technique)

Bench reconstruction was performed on the back table using the York Plastic technique. After the inspection of the injured RCVC, irregular venous edges were minimally trimmed when necessary in order to preserve the maximum amount of viable caval tissue. In Type 1 injuries, the first reconstructive step consisted of suturing together the upper lateral extremities of the defect (A to B) with a 6–0 nonabsorbable monofilament polypropylene suture, thereby creating the apex of the future reconstruction (Figure 1). The remaining side wings (*C*, *C*′, and *D*), where *B* to *D* corresponded to the residual ostium of the left renal vein, were then elevated to define the Y‐shaped configuration that forms the basis of the York Plastic conduit. Reconstruction was completed with three continuous polypropylene sutures running along the limbs of the *Y*‐shape (AB to *C*, AB to *C*′, and AB to *D*), with the aim of enlarging the venous outflow while preserving a smooth inner surface and avoiding stenotic closure.

In Type 2 injuries, the same reconstructive principle was applied, but the first step was modified because the transection caused loss of right renal venous tissue and disrupted the adjacent left renal vein ostial rim (Figure 2). In these cases, the initial approximation was directed first to the disrupted ostial component (*B* to *B*′), followed by approximation of *A* to BB’, in order to recreate the outflow origin before completing the Y‐shaped reconstruction. The remaining caval flaps were then aligned and sutured in continuity according to the same York Plastic concept, thereby recreating a broad venous cone while compensating for the loss of renal vein tissue. After the completion of the reconstruction, excess IVC tissue was excised as needed so that the final conduit represented the shortest length compatible with a tension‐free venous anastomosis, without redundancy likely to promote torsion or kinking. Final geometry, symmetry, and patency were assessed by visual inspection and gentle flushing before implantation. This distinction between Type 1 and Type 2 injury was therefore not merely descriptive, but informed the first step of reconstruction and the degree to which the native venous outflow origin could be preserved.

### 2.4. Implantation and Posttransplant Surveillance

Kidneys were implanted according to standard institutional practice. Venous outflow was anastomosed end‐to‐side to the iliac vein, and the arterial inflow was reconstructed using the donor aortic patch when available. Postoperative surveillance included Doppler ultrasound and laboratory assessments performed daily during the first postoperative week and then at scheduled follow‐up visits. Doppler review focused on venous patency, peak venous velocity, qualitative turbulence/aliasing, and intrarenal resistive indices. Antithrombotic prophylaxis consisted of subcutaneous enoxaparin sodium (20 mg once daily) for 7 days followed by oral acetylsalicylic acid (100 mg once daily) for up to 12 months, unless contraindicated. This regimen reflected our institutional prophylactic practice for reconstructed venous outflow and was intended to minimize early thrombotic risk while maintaining postoperative safety. Cold ischemia time was recorded in absolute terms and compared descriptively with the institutional average for deceased‐donor right‐kidney transplantation over the same interval.

### 2.5. Outcomes

The primary endpoint was early venous complication after reconstruction (RRV/RCVC thrombosis or stenosis), assessed clinically and by Doppler ultrasound. Secondary endpoints included delayed graft function, graft loss, patient survival, bench reconstruction time, cold ischemia time, and graft function at follow‐up. Renal function was summarized using serum creatinine values at 1, 3, and 12 months when available.

### 2.6. Statistical Analysis

Given the limited sample size, all analyses were descriptive. Categorical variables were summarized as counts and percentages, whereas continuous variables were reported as mean ± standard deviation or median with interquartile range (IQR), as appropriate. No formal hypothesis testing, subgroup comparison, or adjustment for multiple comparisons was performed. No imputation was performed; analyses used available data, and denominators are reported where relevant.

The study is reported in accordance with the STROBE recommendations for observational studies.

## 3. Results

Of the 334 deceased‐donor right kidneys assessed, 17 (5.1%) had procurement‐related RCVC injury. Fifteen were reconstructed and transplanted, whereas 2 were not used because of severe associated arterial and/or parenchymal damage. All 15 reconstructed kidneys were transplanted successfully. No vascular complications, including renal vein thrombosis or stenosis, were observed during the early postoperative period. Bench reconstruction time ranged from 20 to 51 minutes (mean 35 minutes). Median peak venous Doppler velocity during the first postoperative week was 32 cm/s (IQR, 27–39). Venous Doppler findings were interpreted using both quantitative and qualitative criteria, and no focal aliasing, turbulent flow, or focal venous velocity acceleration suggestive of clinically relevant outflow stenosis was observed on serial examinations. Median intrarenal resistive index was 0.67 (IQR, 0.62–0.71). Mean cold ischemia time was 16.4 h (SD 2.5), compared with an institutional mean of 15.8 h (SD 3.6) for deceased‐donor right‐kidney transplantation over the same period. Delayed graft function occurred in 3 recipients (20.0%). Median serum creatinine among functioning grafts was 1.58 mg/dL at 1 month, 1.49 mg/dL at 3 months, and 1.36 mg/dL at 12 months. At the 12‐month landmark, 13 of the 14 surviving recipients had functioning grafts. One recipient died at 3 months from COVID‐19–related pneumonia with a functioning graft. One surviving recipient experienced graft failure and returned to dialysis before 12 months because of antibody‐mediated rejection associated with severe cytomegalovirus infection; graft nephrectomy was subsequently performed on postoperative Day 533. In addition, one patient developed pneumonia associated with mTOR‐inhibitor‐based immunosuppression at 24 months after transplantation; this was successfully treated and graft function remained stable. Descriptive statistics for the study cohort, including organ utilization, early graft function, and renal function at follow‐up, are summarized in Table 1, Part C.

**TABLE 1:** Clinical characteristics, posttransplant outcomes, and descriptive statistical summary of kidneys with procurement‐related renal–caval vein complex injury.

Part A. Baseline, injury pattern, and intraoperative characteristics.										
Case	Tx	Recipient Age/sex	Donor Age/sex	Recipient BMI	Donor BMI	RCVC injury type	Bench surgery (min)	Operative time (min)	Cold ischemia time (h)	Status/note
1	N	N/A	67‐M	N/A	35.2	2	—	—	—	Arterial and parenchymal damage
2	N	N/A	61‐F	N/A	34.0	1	—	—	—	Extensive parenchymal injury
3	Y	58‐F	62‐F	25.2	26.0	1	48	108	14.2	Functioning graft
4	Y	57‐F	55‐M	22.7	28.9	2	51	125	15.1	Functioning graft
5	Y	46‐F	59‐F	21.9	33.4	1	22	137	16.8	DGF; graft loss after AMR/CMV
6	Y	40‐F	56‐M	32.0	26.9	1	46	203	18.7	Functioning graft
7	Y	57‐F	68‐F	24.7	25.4	1	47	142	13.5	Functioning graft
8	Y	63‐M	70‐F	23.4	28.3	2	42	123	17.4	Functioning graft
9	Y	64‐M	72‐M	26.1	34.8	2	24	149	14.9	Death at 3 mo with functioning graft
10	Y	38‐F	57‐F	19.0	26.6	1	35	150	12.8	Functioning graft
11	Y	54‐F	60‐M	24.6	27.7	2	33	134	16.2	Functioning graft
12	Y	51‐M	49‐M	19.4	29.1	1	36	151	15.9	Functioning graft
13	Y	54‐M	62‐M	27.0	35.0	2	29	169	22.1	Functioning graft
14	Y	75‐M	61‐F	22.5	27.2	1	32	155	17.0	Functioning graft
15	Y	61‐M	66‐M	24.8	31.5	2	20	144	19.3	DGF; functioning graft
16	Y	49‐F	58‐F	23.6	30.9	1	28	139	18.2	DGF; functioning graft
17	Y	52‐M	64‐F	28.3	31.9	2	32	146	13.6	Functioning graft

*Part B. Posttransplant outcomes and vascular surveillance*										
Case	DGF	Serum creatinine at Tx (mg/dL)	1 mo	3 mo	6 mo	1 yr	Bleeding	Stenosis	Thrombosis	Outcome/follow‐up note
1	N/A	—	—	—	—	—	N/A	N/A	N/A	Arterial and parenchymal damage
2	N/A	—	—	—	—	—	N/A	N/A	N/A	Extensive parenchymal injury
3	N	5.46	1.73	2.48	2.14	1.36	N	N	N	Functioning graft
4	N	3.24	0.94	1.05	1.07	1.04	N	N	N	Functioning graft
5	Y	11.09	3.00	3.86	4.39	—	N	N	N	DGF; graft loss after AMR/CMV
6	N	8.14	1.30	1.27	1.24	1.28	N	N	N	Functioning graft
7	N	3.90	1.21	1.29	1.24	1.12	N	N	N	Functioning graft
8	N	6.14	1.58	2.08	1.81	1.92	N	N	N	Functioning graft
9	N	6.81	1.64	1.56	—	—	N	N	N	Death at 3 mo with functioning graft
10	N	14.32	1.47	1.49	1.37	1.84	N	N	N	Functioning graft
11	N	11.92	1.28	1.29	1.36	1.04	N	N	N	Functioning graft
12	N	5.98	1.09	1.18	1.23	1.21	N	N	N	Functioning graft
13	N	14.37	1.40	1.49	1.43	1.38	N	N	N	Functioning graft
14	N	7.47	1.58	1.36	1.29	1.54	N	N	N	Functioning graft
15	Y	12.50	2.01	1.74	1.82	1.97	N	N	N	DGF; functioning graft
16	Y	14.95	2.38	1.92	1.69	1.54	N	N	N	DGF; functioning graft
17	N	8.42	1.72	1.52	1.43	1.33	N	N	N	Functioning graft

*Part C. Descriptive statistical summary*										
Domain			Metric					Value		
Study sample			Deceased‐donor right kidneys assessed					334		
Study sample			Kidneys with procurement‐related RCVC injury					17/334 (5.1%)		
Injury pattern			RCVC injury type 1					9/17 (52.9%)		
Injury pattern			RCVC injury type 2					8/17 (47.1%)		
Organ utilization			Reconstructed and transplanted					15/17 (88.2%)		
Early graft function			Delayed graft function (transplanted kidneys)					3/15 (20.0%)		
Vascular events			Bleeding/stenosis/thrombosis					0/0/0		
Twelve‐month outcome			Functioning grafts among recipients surviving to 12 months					13/14 (92.9%)		
Renal function			Median creatinine at 1 month, mg/dL (IQR)					1.58 (1.29–1.73)		
Renal function			Median creatinine at 3 months, mg/dL (IQR)					1.49 (1.29–1.83)		
Renal function			Median creatinine at 12 months, mg/dL (IQR)					1.36 (1.21–1.54); *n* = 13		
Ischemia			Mean cold ischemia time, hours					16.4 ± 2.5		

*Note:* Creatinine values are reported in mg/dL. At the 12‐month landmark, one recipient had died at 3 months with a functioning graft and was excluded from the surviving‐recipient denominator; one surviving recipient had returned to dialysis before 12 months. Part C contains descriptive statistics only; no formal hypothesis testing was performed.

Abbreviations: BMI, body mass index; DGF, delayed graft function; RCVC, renal–caval vein complex; Tx, transplanted.

## 4. Discussion

The main contribution of this expanded series is not the introduction of a new concept, which was already presented in 2023, but a more mature clinical characterization of the York Plastic technique in practice. Specifically, the present study broadens the cohort, extends follow‐up, formalizes a pragmatic RCVC injury‐pattern classification, and supports the reproducibility of the reconstruction in a real‐world deceased‐donor setting. The data should therefore be interpreted as an incremental but clinically relevant feasibility update rather than as definitive proof of superiority over other venous extension strategies.

The right kidney remains particularly vulnerable to procurement‐related injury that may limit transplantation [4]. Although implantation of a short RRV can often be achieved successfully [13], technical difficulty may increase in the presence of deep recipient vessels, obesity, complex venous orientation, or procurement‐related damage [5, 7]. These constraints are especially relevant on the right side because the RRV is shorter and anatomic variability may be greater than on the contralateral side [6, 7]. In this context, preservation of venous caliber at the outflow origin may be more important than simply obtaining additional length.

During multiorgan procurement, the right kidney is routinely recovered with a segment of IVC to facilitate the extension of the RRV without compromising liver retrieval [5, 7]. Because the IVC is relevant to both liver and kidney transplantation, transection must be carefully balanced. Rapid or overly aggressive procurement may result in an inadequate caval cuff, thereby compromising graft usability [14]. When excessive transection involves the RCVC and threatens ostial caliber, conventional transverse closure may be suboptimal because it can create narrowing near the left renal vein orifice [15]. The York Plastic technique was developed for this specific subset of injuries, in which the central problem is loss of venous tissue at or near the outflow origin rather than simple short‐vein length.

Several bench strategies have been described to address a short or injured RRV during deceased‐donor procurement, including IVC‐based cuff or patch reconstructions, stapler‐assisted elongation, cryopreserved vascular graft interposition, internal jugular vein extension, and simple elongation maneuvers [8, 9, 15, 16]. Each technique reflects a different balance of tissue availability, technical complexity, and risk of narrowing, torsion, or kinking. A practical comparison of these reconstructive strategies is provided in Table 2 York Plastic should therefore be considered within this broader reconstructive armamentarium rather than as a universal solution. In particular, it is not the only technique available in the literature for the correction of Type 1 RCVC injuries, in which the native RRV ostium remains preserved and other reconstructive maneuvers may also be appropriate depending on local anatomy and surgeon preference. By contrast, the conceptual and practical strength of York Plastic is most evident in Type 2 injuries, where excessive transection compromises the ostial rim and conventional closure or tubulization is more likely to reduce outflow caliber. In such cases, reshaping the residual IVC into a Y‐configured venous conduit is intended not only to provide additional reach for implantation, but also to recreate a broad outflow cone and preserve luminal geometry.

**TABLE 2:** Comparative overview of right renal vein reconstruction strategies for short or procurement‐injured right kidneys.

Technique	Typical indication	Main advantage	Main limitation	When York Plastic may be preferred or avoided
Simple elongation maneuver	Mild short RRV with usable local tissue	Fast and technically straightforward	Limited gain in caliber and length	Not preferred if the ostial rim is compromised
Classic IVC cuff/tubulization	Short RRV with preserved ostium and adequate caval cuff	Uses local tissue and is familiar to many teams	May narrow the outflow if closure is too tight	York Plastic may better preserve lumen when standard closure risks stenosis
Stapler‐assisted elongation	Selected deceased‐donor cases with favorable cuff geometry	Rapid construction	Dependent on injury pattern and cuff configuration	York Plastic may be better when anatomy is irregular or ostial tissue is lost
Interposition graft	Complex anatomy or inadequate local tissue	Can provide substantial additional length	Requires extra conduit and may add anastomoses	York Plastic avoids a separate graft when local IVC tissue is sufficient
York Plastic	RCVC injury with ostial tissue loss and risk of narrowing after standard closure	Aims to preserve outflow caliber while adding reach	Bench complexity; evidence limited to small series	Preferred in the specific setting of threatened ostial caliber after excessive RCVC transection

*Note.* This table provides a practical comparison of commonly used reconstructive options and highlights the situations in which the York Plastic technique may be preferred or avoided.

Abbreviations: IVC, inferior vena cava; RCVC, renal–caval vein complex; RRV, right renal vein.

No thrombosis was observed in our series, although the sample size remains too small to exclude uncommon complications. Accordingly, our findings should be interpreted as supporting technical feasibility, reproducibility, and short‐term clinical safety rather than demonstrating comparative outcome superiority. The reconstructed IVC segment was trimmed and calibrated to fit recipient anatomy and to minimize redundancy, torsion, or kinking. Serial Doppler surveillance was used to assess patency of the reconstructed conduit, and prophylactic anticoagulation was administered according to institutional practice. Longer follow‐up and larger cohorts will be necessary to better define the true incidence of late stenosis or thrombosis after this reconstruction.

In our experience, 17 of 334 deceased‐donor right kidneys assessed during the study interval (5.1%) showed procurement‐related RCVC injury; 15 of these grafts were reconstructed and transplanted with the York Plastic technique, whereas 2 were not used because concomitant arterial and/or parenchymal injury rendered the grafts unsuitable for transplantation. These data suggest that the technique may have practical value not only as a reconstructive maneuver but also as an organ‐preservation strategy in selected cases. At the same time, we did not include a contemporaneous comparator group of noninjured deceased‐donor right kidneys from the same period, and therefore, our findings should not be interpreted as demonstrating equivalence or superiority relative to standard right‐kidney transplantation. Rather, they support feasibility, reproducibility, and short‐term safety in a selected bench‐salvage setting. Donor BMI was numerically higher in donors of kidneys with Type 2 than Type 1 RCVC injury. Given the small sample size, this finding is descriptive only, and the present cohort does not allow the assessment of whether donor habitus contributed to procurement complexity or injury pattern.

In a nationwide Dutch cohort (2015–2020), discard of procured kidneys was 7.8%; when silent nonutilization before procurement was included, total nonutilization rose to 24.4%, and presumed impaired organ quality accounted for 34.2% of discarded kidneys [17]. Although our study does not directly measure reduction in discard rates, the clinical rationale for salvage reconstruction is aligned with the broader goal of limiting preventable nonuse of otherwise transplantable organs.

Beyond technical considerations, procurement‐related injuries are increasingly recognized as a quality metric. Quality monitoring programs and feedback loops during abdominal organ procurement have been associated with measurable effects on transplant outcomes, supporting standardized training and continuous auditing to reduce avoidable damage [7]. In practical terms, York Plastic may be considered when the graft is otherwise suitable for transplantation, sufficient local venous tissue remains reconstructable, and the expected benefit of salvaging the organ outweighs the additional bench complexity. Conversely, it may be less appropriate when tissue loss is too extensive to maintain a wide outflow, when conduit geometry remains unsatisfactory after reconstruction, or when overall graft quality is already prohibitive for transplantation.

The present study has several limitations. It is a retrospective single‐center experience with a small sample size, limited statistical power, and no contemporaneous control group. Part of the transplanted cohort overlaps with our preliminary AJT 2023 report, although the present manuscript expands that early experience by including additional reconstructed cases, nonreconstructed injured grafts, longer follow‐up, and a more explicit injury‐pattern classification. The Type 1/Type 2 framework was developed pragmatically to describe the relationship between the caval transection plane and the RRV ostium and to guide the initial reconstructive step; it has not been externally validated, and its interobserver agreement and broader clinical utility remain unknown. In addition, the present experience was obtained at a high‐volume transplant center with substantial expertise in complex bench vascular reconstruction. Successful application requires careful assessment of residual caval tissue, preservation of viable venous margins, and judgment regarding final conduit geometry. Reproducibility and safety may therefore depend on local surgical expertise, graft selection, and tissue quality, and generalizability to centers with limited experience in complex bench reconstruction remains uncertain. The evidence should consequently be interpreted primarily as support for feasibility and short‐term safety, while broader claims regarding comparative benefit or long‐term superiority should await larger multicenter analyses.

## 5. Conclusions

In this expanded single‐center series, the York Plastic technique appeared to be a feasible bench reconstruction for selected procurement‐related RCVC injuries involving the RRV outflow. The absence of early thrombosis or stenosis, together with acceptable cold ischemia times and graft function during follow‐up, supports its use as an organ‐preserving option in carefully selected cases. Because these data arise from a small retrospective experience, broader claims regarding comparative benefit or long‐term superiority should await larger multicenter analyses.

## Author Contributions

N.M.R.E. conceived the study, collected and curated data, performed the analysis, and drafted the manuscript. R.G.B. contributed to methodology, data acquisition and interpretation, and critical revision. L.B. and A.A. provided supervision, validated the work, and critically revised the manuscript.

## Funding

This research received no external funding.

## Disclosure

All authors reviewed and approved the final version and accept accountability for the work.

## Ethics Statement

This single‐center retrospective observational study was conducted at the Azienda Ospedaliera Universitaria Integrata di Verona (AOUI Verona) in accordance with the Declaration of Helsinki and used fully anonymized, routinely collected clinical, surgical, and follow‐up data. According to the Standard Operating Procedures of CET‐ASOV (Territorial Ethics Committee for the South‐West Veneto Area), the competent ethics committee for AOUI Verona, retrospective observational studies using anonymized data or data from participants who have consented to personal‐data processing are subject to an institutional acknowledgment process rather than prior full ethics committee review.

## Consent

Written informed consent for the use and publication of clinical data in anonymized form was obtained from all 15 transplant recipients. No donor‐specific identifiable data were collected or analyzed.

## Conflicts of Interest

The authors declare no conflicts of interest.

## Supporting Information

Additional supporting information can be found online in the Supporting Information section.

## Supporting information


**Supporting Information** Supporting Figure S1. Flow diagram of procurement‐related RCVC injury in deceased‐donor right kidneys. Among 334 deceased‐donor right kidneys assessed during the study period, 17 (5.1%) sustained a procurement‐related RCVC injury. Of these, 9 were classified as Type 1 and 8 as Type 2 injuries. Fifteen kidneys underwent venous reconstruction with the York Plastic technique and were transplanted, whereas 2 were not transplanted because of associated severe graft damage. At the 12‐month landmark, 13 of the 14 surviving recipients had functioning grafts. One recipient died at 3 months with a functioning graft, and one surviving recipient experienced graft failure and returned to dialysis before 12 months; graft nephrectomy was performed later during follow‐up.

## Data Availability

The data supporting the findings of this study are available from the corresponding author upon reasonable request.
