# Supplementary material for: Managing the Loss of Right Renal Venous Tissue in Deceased‐Donor Organ Procurement
Source: J Transplant. 2026 Sep 2;2026:3588832. doi: 10.1155/joot/3588832 (PMC13537103; doi:10.1155/joot/3588832)
Supplement: Supplementary file 1 [file JOOT-2026-3588832-s001.docx]

Figure S1. Flow diagram of procurement-related RCVC injury in deceased-donor right kidneys. Among 334 deceased-donor right kidneys assessed during the study period, 17 (5.1%) sustained a procurement-related renal-caval vein complex (RCVC) injury. Of these, 9 were classified as type 1 and 8 as type 2 injuries. Fifteen kidneys underwent venous reconstruction with the york plastic technique and were transplanted, whereas 2 were not transplanted because of associated severe graft damage. At the 12-month landmark, 13 of the 14 surviving recipients had functioning grafts. One recipient died at 3 months with a functioning graft, and one surviving recipient experienced graft failure and returned to dialysis before 12 months; graft nephrectomy was performed later during follow-up.
